# Plasmonic Imaging of Tuning Electron Tunneling Mediated by a Molecular Monolayer

**DOI:** 10.1021/jacsau.1c00292

**Published:** 2021-08-06

**Authors:** Zixiao Wang, Ruihong Liu, Hong-Yuan Chen, Hui Wang

**Affiliations:** † State Key Laboratory of Analytical Chemistry for Life Science, School of Chemistry and Chemical Engineering, Nanjing University, Nanjing 210023, China; ‡ Zhengzhou Tobacco Research Institute of CNTC, Zhengzhou 450001, China

**Keywords:** electron tunneling, plasmonic imaging, tunneling decay constant, molecular monolayer, equivalent circuit model

## Abstract

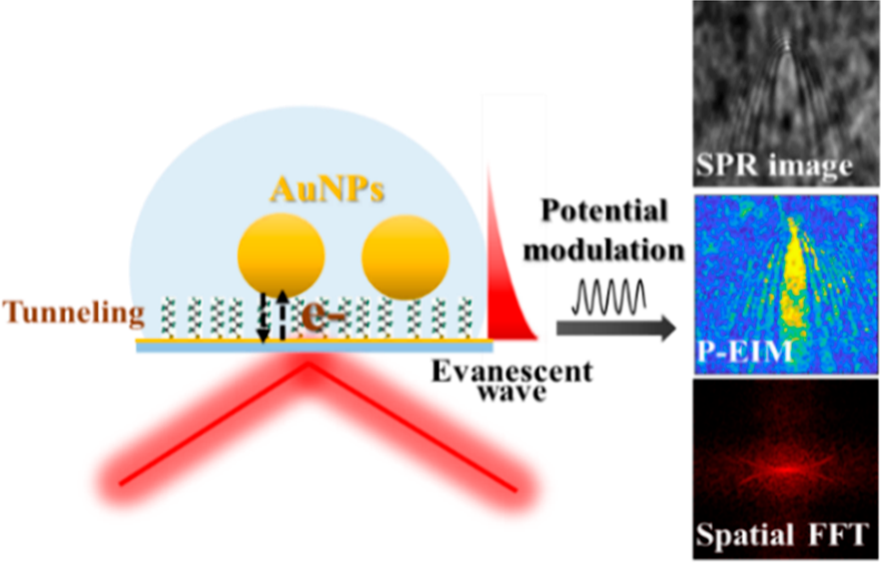

Probing and tuning the electron tunneling in metal electrode–insulator–metal nanoparticle systems provide a unique vision for understanding the fundamental mechanism of electrochemistry and broadening the horizon in practical applications of molecular electronics in many electrochemical systems. Here we report a plasmonic imaging technique to monitor the local double-layer charging of individual Au nanoparticles deposited on gold electrode separated by monolayer of *n*-alkanethiol molecules. The thickness of molecular monolayer tunes the tunneling kinetics and conductivity, which predicts the heterogeneous behavior on the modified electrode surface for different electrochemical systems. We studied the distance dependence of the electron tunneling and double layer charging processes by a plasmonic-based electrical impedance microscopy. By performing fast Fourier transform analysis of the recorded plasmonic image sequences, we can quantify the interfacial impedance of single nanoparticles and the tunneling decay constant of molecular layer. We further observed the electron neutralization dynamics during single-nanoparticle collisions on different surfaces. This optical readout of electron tunneling demonstrates an imaging approach to determine the electrical properties of metal electrode–insulator–metal nanoparticle systems, which include the electron tunneling mechanism and local impedance.

## Introduction

The molecule-level electron tunneling process between a metal electrode and metal nanoparticles mediated by the electrical organic or inorganic insulating layer is a widely discussed topic with fundamental significance in many electrochemical systems.^1−5^ This well-defined methodology, which is proposed for treating double-layer charging current densities in metal electrode–insulator–metal nanoparticle sandwich systems, provides detailed information for electron tunneling mechanism.^6,7^ Another essential application of this nanoscale system is to advance the frontier of fundamental electrochemistry, which shows the capability for the demonstration of molecular electronics and investigation of electrical parameters for molecular monolayer.^8−11^ Furthermore, probing local electron tunneling events between individual nanoparticles and electrodes is still a challenge.

The electron tunneling process between metal nanoparticles and electrode surfaces with hierarchical architectures incorporating self-assembling molecular systems has been studied by many conventional electrochemical techniques to obtain the current of entire electrode.^2,12−14^ These techniques evaluate the average properties of whole electrode, which are limited to obtaining the heterogeneous information at single entity level of nanomaterials and the details of electron tunneling between individual nanoparticles and electrode. Current techniques for measuring local surface charge density and electron transport mechanism of single nanomaterials have been developed. Two main categories stand out: one is probe-based microscopy, including scanning tunneling microscopy, scanning electrochemical impedance microscopy, scanning electrochemical cell microscopy, and conducting atomic force microscopy;^15−17^ the other one is optical approaches without specific probes, such as dark-field microscopy and fluorescence microscopy.^18−21^ The redox mapping of light-addressable electrochemistry is an emerging platform for electrochemical analysis without resorting to the mechanical movement of a probe.^22,23^ These techniques reveal high spatial resolution, which are sensitive to the variation in local charge density on electrode surfaces. Whereas, the lack of temporal resolution and tedious preparation processes of certain probes limit the direct monitoring and tuning of a single entity on the electrode.

In light of the above analysis, the molecular monolayer is imported to regulate tunneling dynamics in the nonfaradaic process or electron transfer in redox transitions.^3,12,24^ Gooding and co-authors have shown that the nanoparticle-mediated organic monolayer system allows efficient electron transfer between redox species in solution and the electrode.^14^ The capacitance of the self-assembled monolayer (SAM) with a good alignment coated on electrode surface can be affected by applied potential. Bueno et al. proposed the detailed theoretical basis and experimental results of mapping the ionic fingerprints of molecular monolayer.^25−27^ These studies provided a complete picture of all the properties of the SAM-modified electrode system and specifically identified the energetic barriers associated with ionic penetration.

In this work, we studied the distance dependence of electron tunneling and double-layer charging processes between individual Au nanoparticles and gold electrodes mediated by a self-assembled monolayer (SAM) with different molecular lengths. We demonstrated the capability of plasmonic-based electrical impedance microscopy (P-EIM) to optically readout local double-layer charging dynamics of individual nanoparticles deposited on gold electrode, which also could be defined as a plasmonic sensing substrate. It provided a sophisticated understanding of electron tunneling mechanism and uncovered the heterogeneity of a single entity for fundamental study of electrochemistry. Furthermore, this approach opens up a promising way to evaluate the performance of organic-monolayer functionalized system and brings various practical applications in photocatalysis, electrocatalysis, and energy transfer in ionic batteries. Inspired by the work of a surface bubble promoting electrochemical reactions,^28^ this technique can also be applied to study the redox activity of different micronanointerfaces.

## Results and Discussion

**Figure 1:**
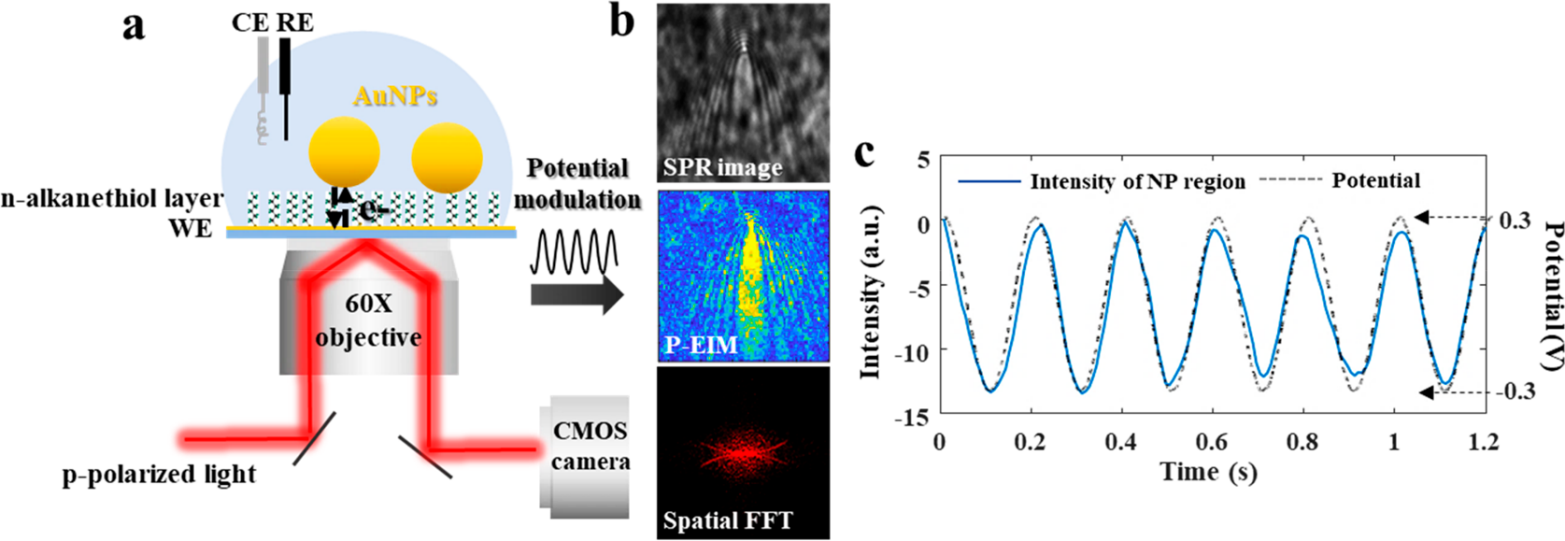
Illustration of experimental setup and mechanism of the plasmonic-based electrical impedance imaging technique. (a) Simultaneous electrochemical measurement and plasmonic imaging setup. (b) Plasmonic image (DC component), the corresponding P-EIM image (AC component), and the spatial fast Fourier transfer (FFT) obtained from the P-EIM image of a single AuNP on the 1-octanethiol (C8)-modified gold electrode. (c) Plasmonic signal (blue curve) of a single AuNP region on the C8-modified gold electrode in b and the synchronous potential modulation (gray dashed curve) versus time. Frame rate: 100 fps.

In this work, we probe the electron tunneling and local double-layer charging processes between single Au nanoparticles (AuNPs) and a gold electrode mediated by a well-assembled *n*-alkanethiol monolayer with the P-EIM imaging technique.^29−31^ The plasmonic-based imaging technique has been developed to map local current density of different nanomaterials, which associates the changes of faradic current and double-layer charging current.^31−34^ By applying sine-wave potential modulation, we introduced the fast Fourier transform (FFT) to analyze the time-lapsed plasmonic images and obtained the DC component (plasmonic image at 0 Hz) and AC component (P-EIM image at modulated frequency) information on individual AuNPs. Figure 1a illustrates the experimental setup and basic working principle of the P-EIM technique, showing AuNPs placed on a gold electrode via an insulating layer of *n*-alkanethiol imaged by a 60× oil immersion objective with a high numerical aperture (NA = 1.49) of an inverted optical microscopy (more details in Materials and Methods). The p-polarized incident light from a superluminescent diode (SLED) was directed onto a 47 nm Au covered glass to excite surface plasmons, and the reflected light was captured by a complementary metal oxide semiconductor (CMOS) camera with low noise level to obtain the time-lapsed plasmonic images. A typical three-electrode system was inserted for potential modulation, where the gold chip was served as a working electrode (WE), whereas the Ag/AgCl wire and Pt foil served as the reference (RE) and counter electrodes (CE), respectively. To uncover the electron tunneling dynamics and local impedance information, we applied a sinusoidal potential generated from a function generator to the system via a bipotentiostat.

Plasmonic imaging is extremely sensitive to the variation in local charge density, which can affect the optical property of electrode (dielectric constant, *ε*_m_). By capturing the time-lapsed plasmonic intensity change, the electron tunneling current (*i*_tun_), which is equal to the double-layer charging current (*i*_dl_), can be extracted and lead to P-EIM. The local double-layer charging current density (*J*_d_) is directly related to the plasmonic image intensity (*I*_p_) according to
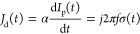
1In eq 1, α is a coefficient that can be calibrated experimentally or calculated theoretically by the Drude model (free electron gas model). Parameter *f* is the frequency of applied potential, and σ is local charge density. The change of plasmonic angle (Δθ) by potential modulation is proportional to Δσ, which is externally manifested as the AC component of plasmonic imaging at certain modulation frequency. The DC component can be displayed simultaneously to present conventional plasmonic images at 0 Hz. Figure 1b shows the plasmonic image, the *in situ* P-EIM image, and the corresponding spatial fast Fourier transfer (FFT) of the P-EIM image for a single AuNP (diameter = 100 nm, the size characterization by scanning electron microscopy is shown in Supporting Information S7) on the 1-octanethiol (C8)-modified gold electrode. The characteristic parabolic pattern of plasmonic imaging was minutely explained in the previous literature.^35^ The FFT of P-EIM image in *k*-space shows the distinctive bright semicircles that are from the scattering of propagated plasmonic waves, which revolves around AuNPs in the real space P-EIM images.^33,34^ The large background signal of gold electrode during charging–discharging process can be blocked by the *n*-alkanethiol modification to reveal a clean background. This further increases the contrast between AuNPs and electrode, and provides the possibility to extract the signal from AuNP region. According we described above (*i*_tun_ = *i*_dl_), the P-EIM image reveals the intensity change related to local charge density in the particle region, which shows a bright pattern of AuNP region in Figure 1b. Figure 1c shows the plasmonic intensity change of single AuNP region on the C8-modified gold electrode in Figure 1b and the synchronous sine-wave potential applied to system.

**Figure 2:**
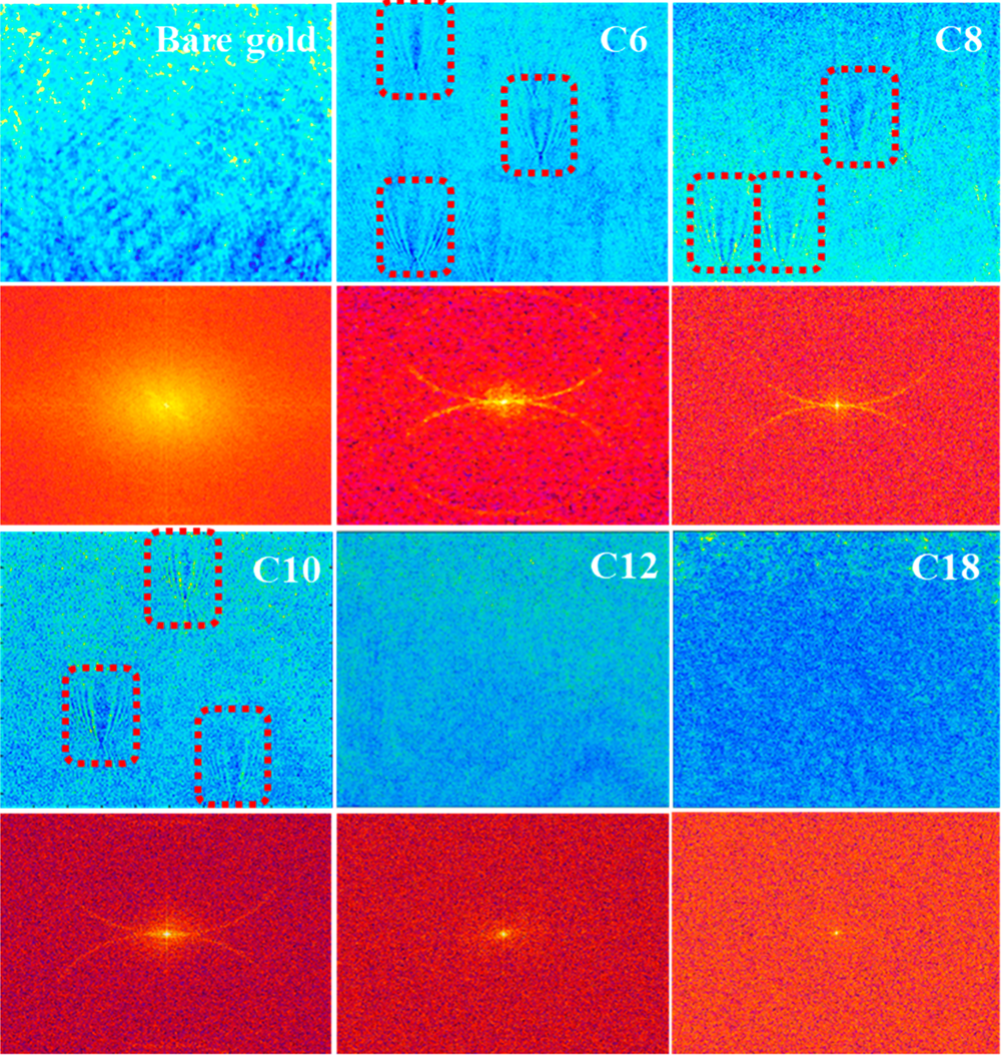
P-EIM images for individual AuNPs (diameter = 100 nm) deposited on the local electrode surface, and the corresponding spatial FFT for a single AuNP region on different surfaces (bare gold or C6-, C8-, C10-, C12-, and C18-modified gold electrodes), revealing a pair of semicircles. The image contrast for P-EIM represents local current densities of the electrode. The distinctive bright semicircles are extracted from the spatial FFT of P-EIM image in *k*-space, which are induced by the scattering of propagated plasmonic waves. The frequency of sine-wave potential modulation is 5 Hz and the amplitude is ±0.3 V. The red dashed squares point out accurate locations of individual AuNPs on the substrate.

To study the electron tunneling process between AuNPs and gold electrodes, we performed a double-layer charging experiment on a gold electrode modified with different lengths of *n*-alkanethiol within a relatively small potential region to avoid the faradic current. The capacitance obtained from cyclic voltammograms decreased with the length of insulating monolayer (more details in Supporting Information S1) by cycling potential within the double-layer charging regime. The roughness of different alkanethiol modified gold electrodes was determined by the contact-mode atomic force microscopy in deionized water (Supporting Information S6). We tuned the distance between AuNPs and the gold electrode by inserting different *n*-alkanethiols and visualized it by P-EIM in Figure 2 and Figure S2. For a bare gold electrode without any modification, the deposited AuNPs were identified as part of the gold electrode and presented no obvious contrast from P-EIM images at the modulation frequency (5 Hz). The amplitudes of the electro-optical signal for AuNPs and gold substrate are nearly the same. The spatial FFT from the P-EIM image reveals no characterized scattering signal when both AuNPs and background (bare gold electrode) are charged. The FFT signal of the whole imaging region shown in Figure S3 brings out the average charging signal including AuNPs and gold electrode. For thin *n*-alkanethiol modification (*n* = 6, 8, and 10), the double-layer charging of the electrode surface without AuNPs was blocked by the insulating monolayer, and the AuNP regions showed obvious contrast in P-EIM images because of the electron tunneling and double-layer charging processes. As the molecular thickness continues to increase, the P-EIM contrast decreases and finally disappears, which further confirms that the local tunneling current can be hindered and tuned at single-nanoparticle level in Figure 2 and Figure S2. For thick *n*-alkanethiol modification (*n* = 12 and 18), the electron tunneling process was fully cut off, and the P-EIM image just displayed the fluctuations in noise level. This level of detailed information cannot be obtained with the ensemble measurements.

**Figure 3:**
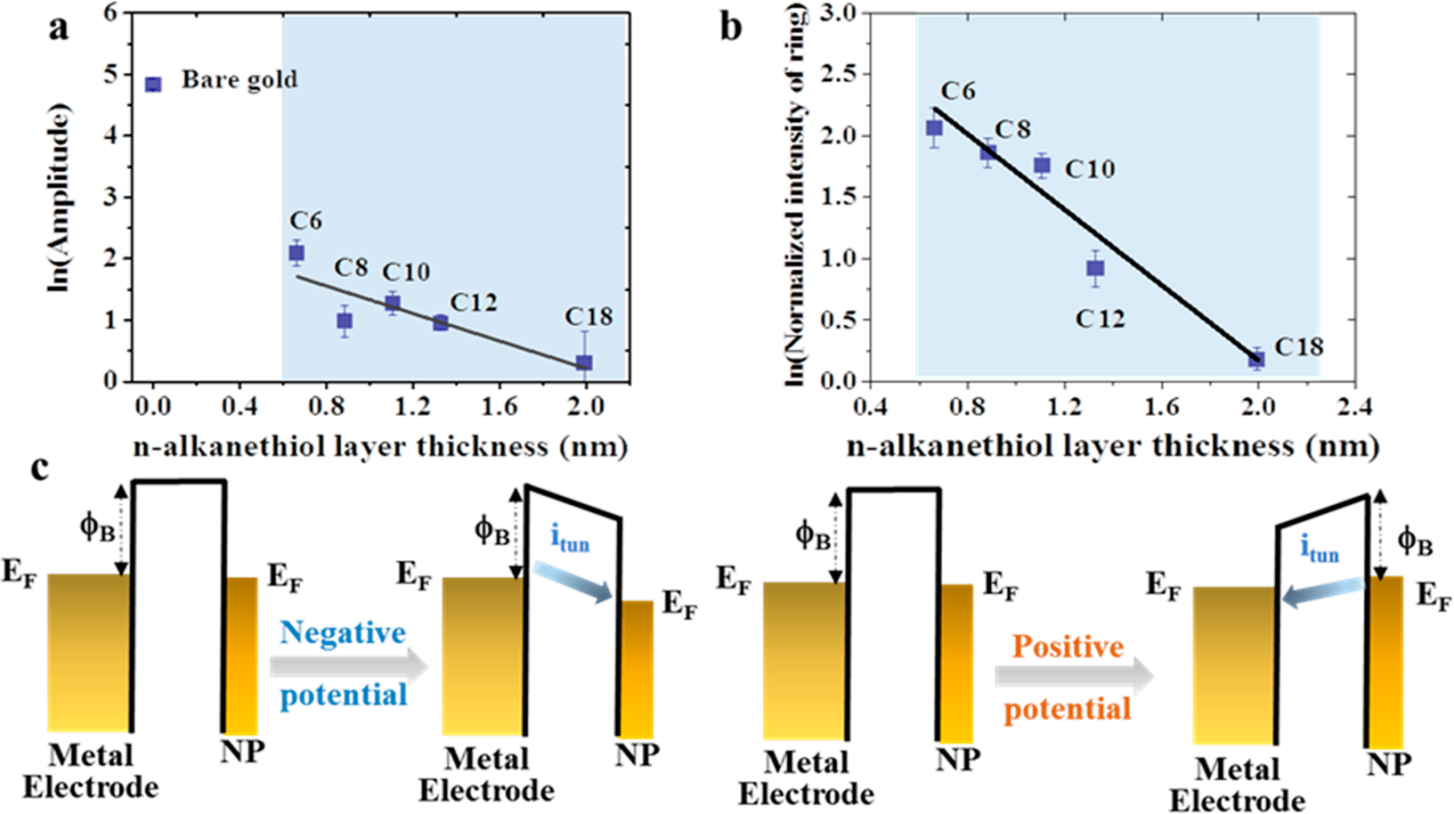
(a) Semilogarithmic plot of the optical amplitude for the AuNP region obtained from P-EIM images versus the thickness of *n*-alkanethiol monolayer. The black curve shows the linear fitting result. (b) Semilogarithmic plot of normalized intensity obtained from the spatial FFT ring versus the *n*-alkanethiol monolayer thickness. The black curve shows the linear fitting result. (c) General schematic tunneling models for metal electrode–insulator–metal nanoparticle system uncovering the tilt of tunneling barrier between the Fermi levels of gold electrode and AuNPs by applying positive or negative potential. The electrolyte is 0.1 M NaF solution, and the reference electrode is Ag/AgCl.

The semilogarithmic plot of the plasmonic intensity change obtained from Figure 2 versus the thickness of the *n*-alkanethiol monolayer is presented in Figure 3a. In the metal electrode–insulator–metal nanoparticle system, the electron tunneling rate (*k*) can be given by
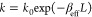
2where *k*_0_ is a constant, *β*_eff_ is the effective tunneling decay constant for the metal electrode–insulator–metal nanoparticle system, and *L* is the tunneling distance determined by the thickness of *n*-alkanethiol monolayer modified gold electrode. The tunneling distances of different alkanethiol modifications were extracted from the capacitance measurements shown in Figure S1. We have demonstrated in our previous work that the tunneling current is equal to the local refractive index change in plasmonic images, which is associated with charge density variation.^36^ From the linear dependence in Figure 3a, the slope *β*_eff_ is 1.1 ± 0.1 nm^–1^ for different molecular lengths. Figure 3b reveals the semilogarithmic plot of normalized intensity change (*I*_NP_–*I*_Background_, more details in Supporting Information S4) for the spatial FFT obtained from Figure 2 versus the thickness of insulating monolayer. After applying linear fitting, the slope *β*_eff_ is 1.5 ± 0.1 nm^–1^, which is relatively larger and more accurate revealing a pure tunneling signal from AuNPs. The smaller β obtained from P-EIM images is attributed to the background signal such as pinholes and morphological variations in the molecular layer, which is not fully subtracted.^36^ To understand the tunneling decay constant β in our system, we present the energy diagrams of charging and discharging processes in Figure 3c. By applying modulation potential to the metal electrode–insulator–metal nanoparticle system, the tunneling barriers (*ϕ*_B_) between the Fermi level (*E*_f_) of AuNPs and electrode in the tilting and tunneling processes are in reverse directions, which jointly result in the small decay constant we obtained. For a metal nanoparticle in contact with the electrolyte, there is an activation barrier corresponding to the reorganization of the embedding medium upon changing the charge state of nanoparticle, which can be described by the Marcus–Gerischer–Morrison theory. Recent research also uncovered similar results for nanoparticle-mediated electron transfer at the sandwich structure (metal/insulator/metal).^1^

**Figure 4:**
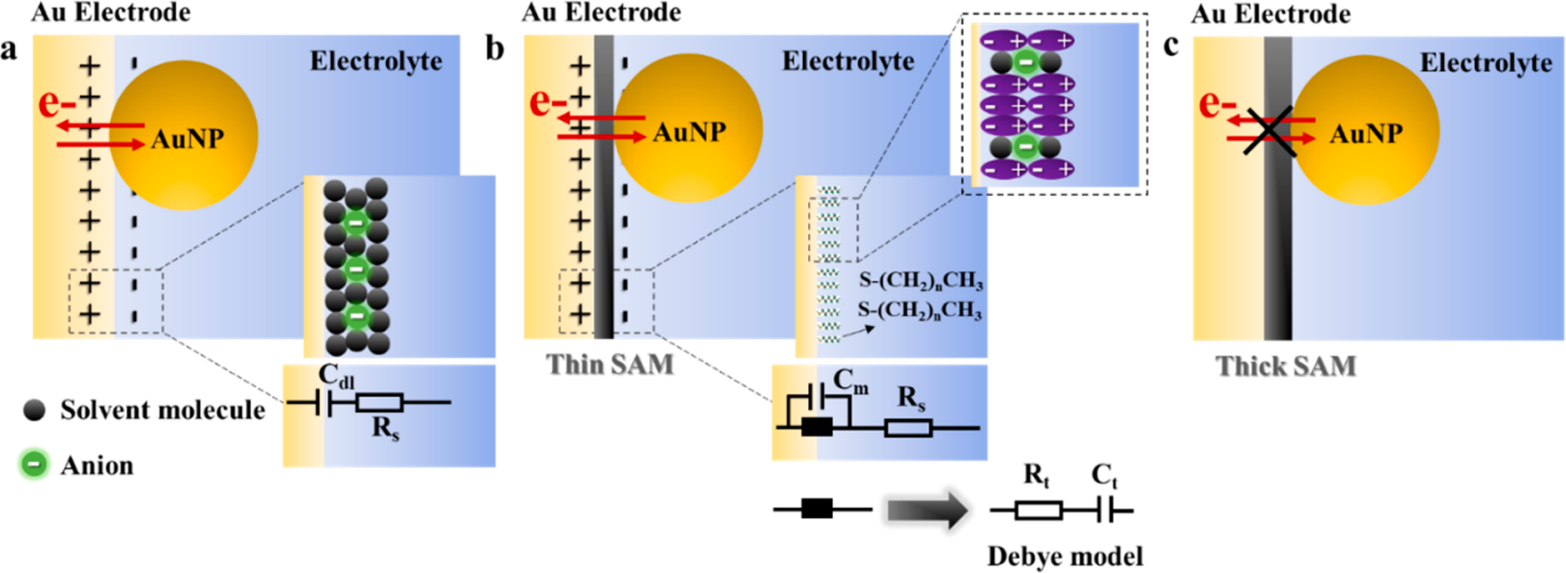
(a) Equivalent circuit representations of AuNPs on a bare gold electrode directly exposed to an electrochemical inert electrolyte solution summarizing the contributions from nonfaradaic processes. In this model, a rigid layer comprising solvent molecules and solvated ions exists and the electrochemical double-layer capacitance is *c*_dl_. (b) For thin SAM modification, the dielectric model was proposed for the dipolar/ionic relaxation and the ingress/trapping described by the classic Debye equivalent circuit. The negatively charged *n*-alkanethiol headgroup links with the gold electrode, and the dominant alkane-S (δ^+^- δ^–^) dipoles exist with solvated ions. The resultant capacitance in electrolyte media is the series capacitance (*C*_m_), which connects in parallel with a black box element (series resistive and conductive terms of ionic dipolar SAM: *R*_*t*_ and *C*_*t*_). For optical imaging, the background of the gold electrode induced by the potential modulation can be blocked with the insulating SAM monolayer. The electrons can tunnel between AuNPs and the electrode to trigger the charging–discharging process of individual AuNPs and make the nanoparticles visualized by P-EIM simultaneously. (c) For thick SAM modification, the electron tunneling process between nanoparticles and electrodes is cut off because of the thick insulating layer. There is no image contrast between AuNPs and background during potential modulation.

The *n*-alkanethiol-modified gold electrode can be modeled by equivalent circuits with several electrical components, which is minutely described by Bueno et al.^25,27,37^ In light of the above study, the electrolyte (0.1 M NaF solution) has a resistive effect (*R*_s_), and the electrochemical double layer formed on the interface between gold electrode and solution has a capacitance (*C*_dl_) in Figure 4a for AuNPs directly deposited on bare gold electrode in our system. The distribution of point ions embedded in a solvent dielectric continuum above a perfectly conducting gold electrode can be determined by electrostatics and statistical mechanics.^26^ An ideal SAM modification without any defects would present no capacitance or resistance. Although real molecular layers are rich with different influence factors.^38^ For thin SAM modification with *n*-alkanethiol (*n* = 6, 8 and 10), the equivalent circuit of the system can be represented by the Debye model^39^ in Figure 4b, and the background of the gold electrode induced by the potential modulation is blocked. In this model, the orientation of inherent dipoles pointing down toward the gold electrode surface is responsive and can be expressed by a generic black-box term parallel to high-frequency capacitance^25,26^*C*_m_. The free electrons can tunnel between AuNPs and the gold electrode via an insulating layer to trigger the charging–discharging process of individual AuNPs and make the nanoparticles visualized by P-EIM simultaneously. For thick SAM modification with *n*-alkanethiol (*n* = 12 and 18), the electron tunneling process between nanoparticles and electrode is fully hindered because of the thick insulating layer beyond the tunneling regime (Figure 4c). There is no image contrast and intensity change between AuNPs and the electrode during potential modulation.

**Figure 5:**
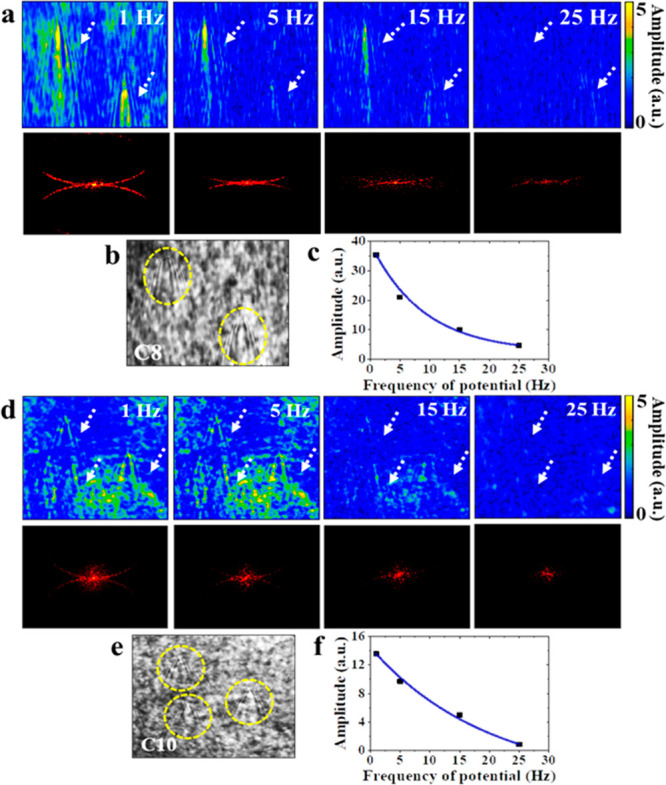
(a) P-EIM images (AC component) and the corresponding spatial FFT of single AuNPs (diameter = 100 nm) on the C8-modified surfaces versus modulation frequencies of applied potential. The white arrows point out the location of nanoparticles. (b) Plasmonic image (DC component) of two AuNPs (yellow dashed cycles) on the C8-modified gold surface. (c) FFT amplitude of the AuNP region versus the modulation frequencies of applied potential. (d) P-EIM images and spatial FFT of single AuNPs on C10-modified surfaces versus modulation frequencies of the applied potential. The white arrows point out the location of nanoparticles. (e) Plasmonic image of three AuNPs (yellow dashed cycles) on the C10-modified gold surface. (f) FFT amplitude of AuNP region versus modulation frequency of applied potential. Modulation amplitude is ±0.3 V. The electrolyte is 0.1 M NaF solution, and the reference electrode is Ag/AgCl. Frame rate: 100 fps.

Based on equivalent circuit models, the intensity change of P-EIM is related to the amplitude and frequency of applied modulation potential. To further demonstrate the above relationship, we performed the P-EIM experiments with the potential modulation of different amplitudes and frequencies. Figure S5 shows the P-EIM images of single AuNP on the C8-modified surface at 5 Hz with different modulation potentials. The contrast increases as the amplitude of potential increases because of the proportional dependence between Δ*V* and Δθ. Furthermore, we measured intensity change of P-EIM images and the corresponding spatial FFT semicycles of different frequencies in Figure 5. For both C8- and C10-modified surfaces, we increased the frequency from 1 to 25 Hz, and the intensity change in single deposited AuNPs decreased with the fixed amplitude (±0.3 V) because of the decrease in tunneling current.

**Figure 6:**
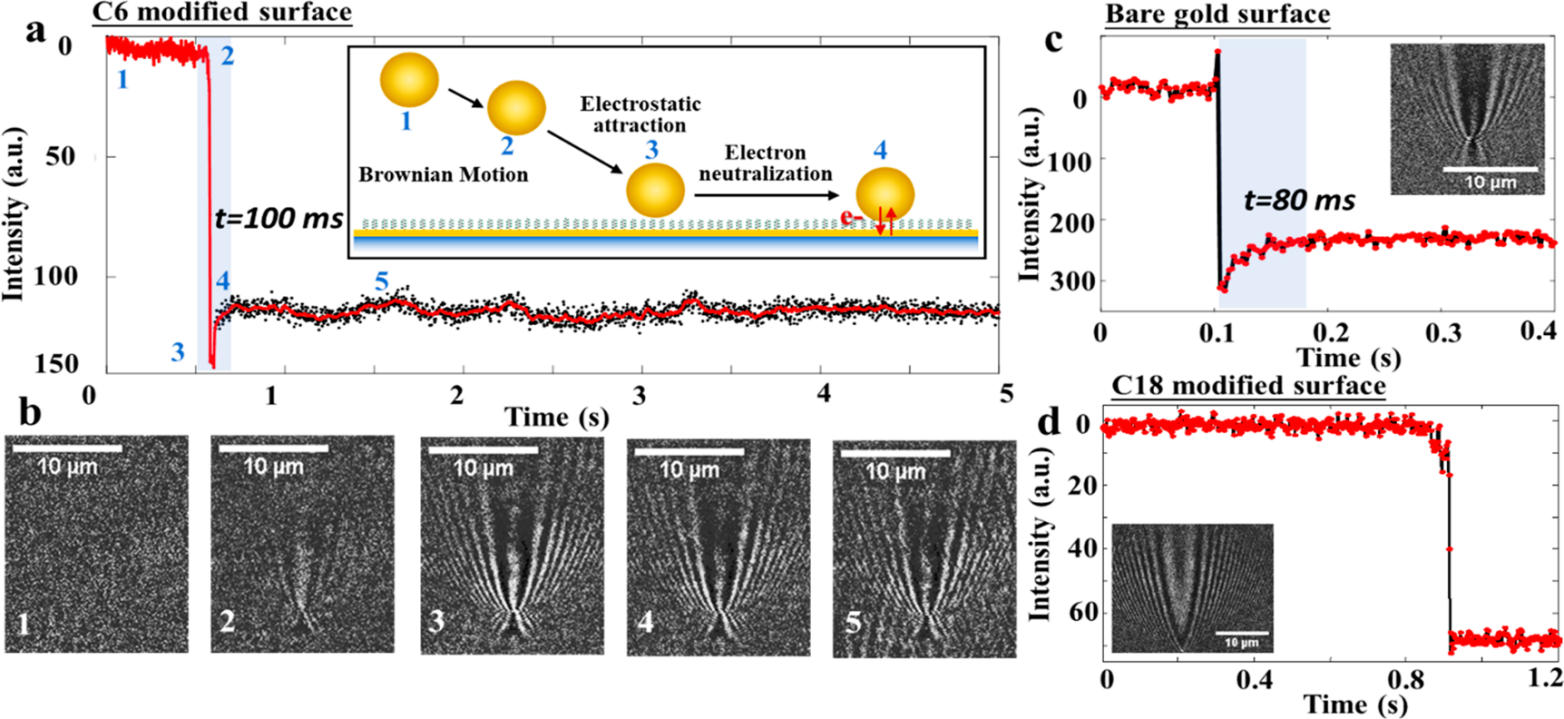
Electron neutralization dynamics during individual nanoparticle collision on different surfaces. (a) Time-lapsed plasmonic intensity curve during a single AuNP hitting the C6 modified surface. The inset illustrates a step-by-step process. The time constant for the electron neutralization process is ∼100 ms. (b) The snapshots of collision process by plasmonic imaging corresponding to different steps in a. (c) Time-lapsed plasmonic intensity curve during a single AuNP hitting the bare gold surface. The time constant for electron neutralization process is ∼80 ms. (d) Time-lapsed plasmonic intensity curve during a single AuNP hitting the C18-modified surface. Frame rate: 438 fps.

To further verify the theoretical model in Figure 4, we explored the instantaneous process of a single-nanoparticle collision on different surfaces with a high frame rate. During the Brownian motion process, single AuNP gets close to the substrate and attaches to surface because of the electrostatic attraction. Although the AuNP just collides with surface, the optical intensity decreases slightly and reveals a kinetic process on bare gold or thin SAM-modified surfaces in Figure 6, which can be attributed to the electron neutralization between AuNP and substrate. The time constant for above process on the bare gold surface is relatively faster than that on the C6-modified surface because of molecular insertion. For bare gold surface, the time constant may be affected by the citrate capping layer around single AuNPs during collision process, whereas the thick SAM modification cuts off the electron neutralization process between AuNP and substrate, and the collision process on the C18-modified surface shows only the hitting signal in Figure 6d. Statistical analysis of the time constant for different surfaces reveals fast (∼76 ms) and slow (∼164 ms) electron neutralization dynamics for bare gold and C6-modified surfaces, respectively (Supporting Information S8).By applying potential, the Simmons model^40,41^, where α = 0.65, *ϕ*_B_ = 1.43 eV (this is determined from the conductance of Au–molecule–Au system)^42,43^ and ζ = 0.5 for uniform tilting of the barrier^8^ in Figure 3c. The above model is expected to show a smaller tunneling decay constant *β*_eff_ observed in the plasmonic imaging method and will detail the inner interaction of metal electrode–molecule–metal nanoparticle system. The optical readout measurement for the tunneling process focused on the tunneling property of a local insulating molecular layer modified on a gold substrate. The decay constant obtained from the optical system reveals the property of the insulating layer, including the pinholes and morphology variations of the modified thin film. The pinhole and deformation of the molecular layer contribute to the distribution of the decay constant. Correct prediction of the decay constant needs to consider the interactions between AuNPs with an *n*-alkanethiol monolayer and detailed information on the sandwich system, which can affect the actual nanoscale gap and mutual effect between AuNPs and gold electrodes.

## Conclusions

In this work, we have demonstrated an optical technique combined with electrochemical measurement to probe the electron tunneling mechanism and local impedance characterization of a molecular monolayer at single nanoparticle level. By tuning the distance between AuNPs and gold electrode via *n*-alkanethiol with different molecular lengths, we captured the DC and AC components of plasmonic image for individual AuNPs from tunneling regime to fully hindered regime, which was further validated by the intensity change of spatial FFT semicycles of P-EIM images. The small tunneling decay constant from P-EIM is attributed to the lowering of tunneling barrier height at positive or negative voltages, deformation variations in the modified monolayer, and the interactions between AuNPs with an *n*-alkanethiol monolayer. They can affect the actual distance between AuNPs and gold electrodes. The results elucidate the equivalent circuit model for a metal electrode–insulator–metal nanoparticle to fully understand and evaluate various aspects of theoretical estimations. It provides important insight into the design and characteristic of analytical devices and molecular electronics, which can deepen the understanding of fundamental electrochemistry and expand the applications in electrochemical sensing, electrochemical energy conversion, and bioelectrochemistry. The distortion effect of the plasmonic pattern and the limitations of electrode materials for requirements to exploit surface plasmon resonance effects restrain the development of this technique and still deserve further study.

## Materials and Methods

### Chemicals

A bare Au nanoparticle (100 nm in diameter) aqueous solution was purchased from Nanopartz. N-Alkanethiols (*n* = 6, 8, 10, 12, and 18) were purchased from Aladdin Industrial Corporation. NaF (powder) was purchased from Sigma-Aldrich and diluted in deionized water. All aqueous solutions were dissolved in deionized water (18 MΩ·cm^−1^, Milli-Q, Millipore Corp.). All experiments were conducted at room temperature.

### Sample Preparation

#### Preparation of Bare Gold Electrodes

The gold films were prepared by depositing a 2 nm thick chromium (Cr) layer and a 47 nm thick gold layer on 22 mm × 22 mm glass microscope coverslips (BK-7) by using a magnetron cosputtering technique. The Cr layer was introduced for increasing the adhesion between the cover-glass and the gold layer. The gold films were treated with a H_2_ flame to remove the surface contaminations before performing each experiment.

#### Preparation of *n*-Alkanethiol-Modified Gold Electrodes

Seven microliters (1-hexanethiol, C6), 8.8 μL (1-octanethiol, C8), 10.7 μL (1-decanethiol, C10), 12.2 μL (1-dodecanethiol, C12), and 13.5 μL (1-tetradecanethiol, C18) were dissolved in 10 mL of ethanol, respectively, to obtain 5 mM surface modified solutions. The treated gold films were immersed in each *n*-alkanethiol solutions for 12 h to form self-assembled molecule monolayer on the surface, rinsed with ethanol, and dried with nitrogen gas. AFM with contact mode was performed to characterized the roughness of the gold electrode surfaces after modification.

### Electrochemical Measurement

A typical three-electrode system was used to induce the electrochemical reaction on the Au nanoparticles, which contained a gold film (a working electrode in which nanoparticles were attached to a bare gold electrode or mediated with different modification layers), a Ag/AgCl wire (reference electrode), and a Pt coil (counter electrode). The electrochemical Teflon cell was filled with 0.1 M NaF solution for all electrochemical experiments in this work. The AuNP aqueous solution with an initial concentration of 50 mM is diluted with deionized water (V:V = 1:50). Then, 10 μL of diluted solution has been injected into the electrochemical cell, and the individual AuNPs collide with the substrate, contributing to the electrostatic attraction. The cyclic voltammetry of sweeping the potential with the regime of double-layer charging for determining the actual thickness of the *n*-alkanethiol layers and the potential modulation for tuning electron tunneling were performed via a potentiostat (Autolab) or bipotentiostat (ARFDE5, Pine) with a function generator (33521A, Agilent) with different frequencies and amplitudes.

### Optical and Characterized Instrumentation

The plasmonic imaging setup was built on an inverted microscope (Nikon Ti-E) with a 680 nm SLED light source (Q-photonics), a high numerical aperture 60× oil-immersion objective lens (NA = 1.49), and a CMOS camera (ORCA-Flash 4.0, Hamamatsu, Japan) with a low noise level. The p-polarized light was directed onto the gold film mounted on the objective for exciting the plasmonic wave, and the reflected light was captured with the same objective and recorded by the CMOS camera for imaging. The potential modulation applied between the gold film and the reference electrode induced the electron tunneling of the insulated *n*-alkanethiol monolayer and the charging–discharging of individual gold nanoparticles. They can be detected with the plasmonic imaging setup, resulting in a series of plasmonic images synchronized with the applied potentials.

The scanning electron microscopy (SEM) characterization of gold nanoparticles was carried out by JSM-7800F to get the morphology and size distribution. Contact-mode AFM images were captured in 0.1 M NaF solution with silicon nitride tips (Agilent Technologies AFM 5500) and the scanning rate was 0.5 line/s.

### Data Extraction and Processing

Data processing was performed with *Matlab* software and *ImageJ*.

Spatial fast Fourier transform was performed to extract the scattering signals from AuNPs by transforming the plasmonic image with potential modulation into the Fourier (*k*) space, which reveals a pair of characteristic semicircles (2D rings). The intensity of each semicircle provides more accurate determination of the plasmonic image intensity of AuNPs.^34,44^

## Supporting Information Available

Capacitance measurements, P-EIM and AFM images, FFT amplitudes, selected region for signal extraction, SEM measurements, and plasmonic intensity curves (PDF)
