# Supplementary material for: Plasmonic Imaging of Tuning Electron Tunneling Mediated by a Molecular Monolayer
Source: JACS Au. 2021 Aug 6;1(10):1700–7. doi: 10.1021/jacsau.1c00292 (PMC8549056; doi:10.1021/jacsau.1c00292)
Supplement: Supplementary file 1 — au1c00292_si_001.pdf [file au1c00292_si_001.pdf]

## Supporting Information

# Plasmonic Imaging of Tuning Electron Tunneling Mediated by a Molecular Monolayer

Zixiao Wang[a], Ruihong Liu[a,b], Hong-Yuan Chen[a], and Hui Wang[a,*]

[a]State Key Laboratory of Analytical Chemistry for Life Science, School of Chemistry and Chemical Engineering, Nanjing University, Nanjing 210023, China;

[b]Zhengzhou Tobacco Research Institute of CNTC, Zhengzhou 450001, China;

*Corresponding author

## Table of Contents

**S1. Cyclic voltammetry and capacitance measurement of the n-alkanethiol modified gold surfaces**

**S2. The false-color P-EIM images of individual AuNPs on different n-alkanethiol modified gold surfaces**

**S3. The FFT amplitude of whole image for different n-alkanethiol modified gold surfaces**

**S4. The selected region for extracting the signal of spatial FFT rings for different n-alkanethiol modified gold surfaces**

**S5. The P-EIM images of single gold nanoparticle on C8 applied with different amplitudes of potential**

**S6. The AFM image and surface roughness of n-alkanethiol modified gold surfaces**

**S7. The SEM measurement of 100 nm AuNPs**

**S8. Statistical analysis of electron neutralization dynamics during individual nanoparticle collision on different surfaces**

**S1. Cyclic voltammetry and capacitance measurement of the n-alkanethiol modified gold surfaces**

Double layer charging measurement is performed to determine the interfacial capacitance (C) for n-alkanethiol modified gold surfaces (the result of C8 in **Figure S1a**). We measured C for n-alkanethiol with different molecular lengths and plotted 1/C versus the number of carbon atoms (n) in **Figure 1Sb**. The capacitance is determined by the n-alkanethiol monolayer thickness (d) which is given by,

$$1/C=\varepsilon\varepsilon_0^{-1}d+\varepsilon\varepsilon_0^{-1}d_{dl}, \quad (S1)$$

where $\varepsilon_0$ is the permittivity of the free space, ε is the dielectric constant of the n-alkanethiol on gold (ε is a constant from literatures[1,2]) and $d_{dl}$ is double layer thickness of n-alkanethiol. From the slope determined by the linear fitting result in **Figure S1b**, we determined the thickness of n-alkanethiol monolayer between gold nanoparticles and gold substrate in **Figure S1c**.

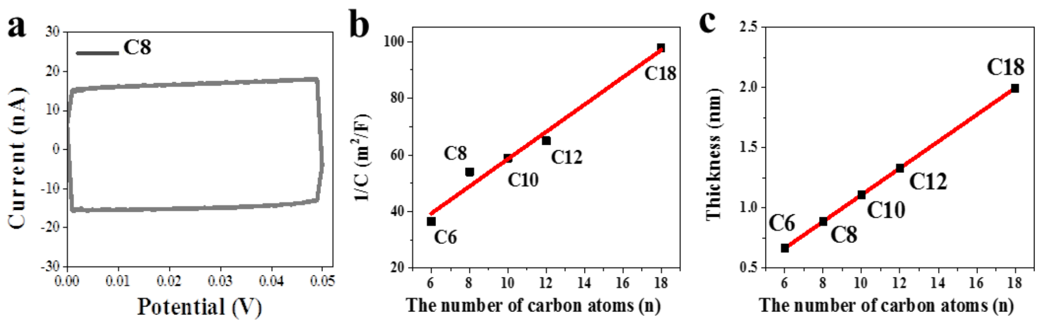


**Figure S1.** (a) The capacitance measurement of C8 modified gold electrode for calculating the thiol thickness (vs. Ag/AgCl). From the capacitance, the thickness of the molecular monolayer can be determined and the results are consistent with the values reported in the references[1,3]. The electrolyte is 0.1 M NaF and the potential cycling rate is 0.01 V/s. (b) The inverse of the capacitance 1/C versus the number of the carbon atoms (n). (c) The thickness of n-alkanethiol versus the number of carbon atoms (n).

**S2. The false-color P-EIM images of individual AuNPs on different n-alkanethiol modified gold surfaces**

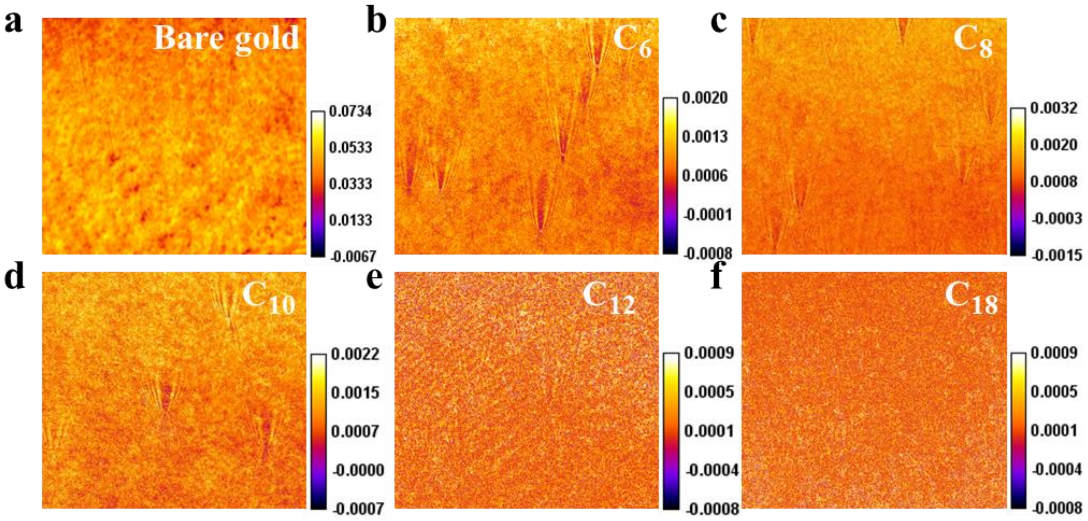


**Figure S2.** The false-color P-EIM images of individual AuNPs (Diameter=100 nm) on bare gold (a), C6 (b), C8 (c), C10 (d), C12 (e) and C18 (f) SAM layer modified gold surfaces. The frequency of sine-wave potential modulation is 5 Hz and the amplitude is ±0.3 V. The electrolyte is 0.1 M NaF solution, and the reference electrode is Ag/AgCl.

## S3. The FFT amplitude of whole image for different n-alkanethiol modified gold surfaces

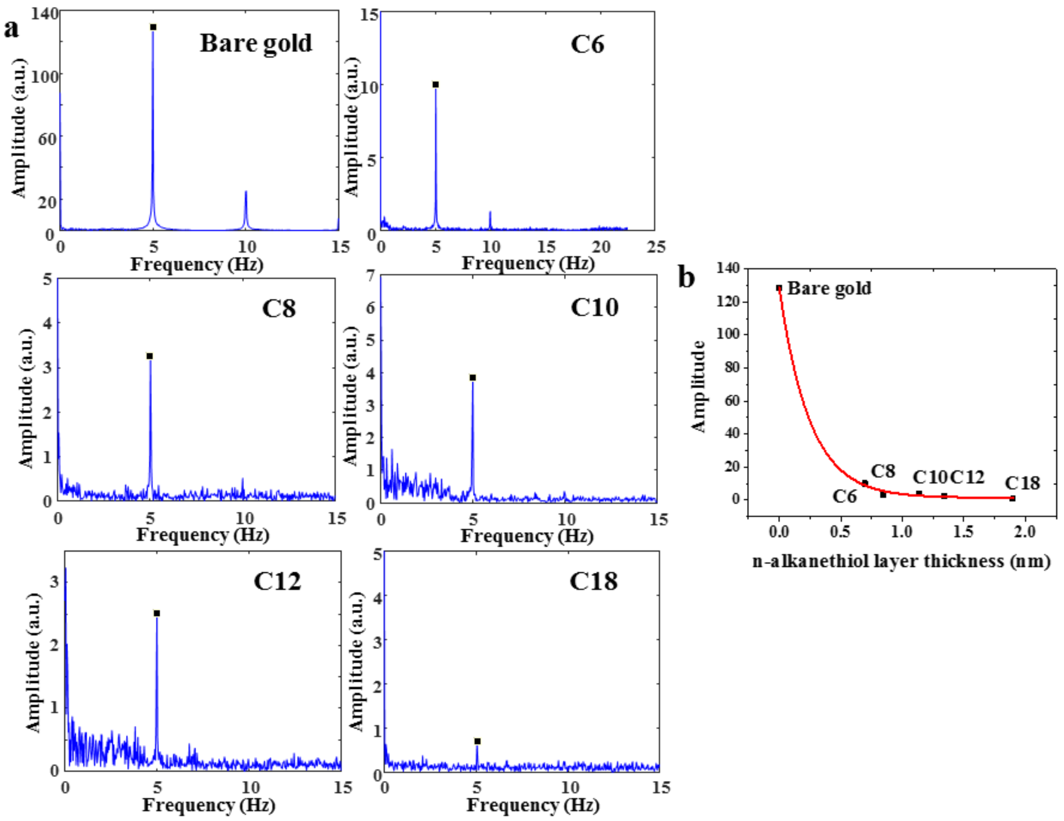


**Figure S3.** (a) The total FFT amplitude (including AuNPs and substrate regions) of plasmonic intensity of the bare gold, C6, C8, C10, C12 and C18 modified gold electrode surfaces. (b) The amplitude obtained from whole plasmonic images versus the thickness of different n-alkanethiol layers.

**S4. The selected region for extracting the signal of spatial FFT rings for different n-alkanethiol modified gold surfaces**

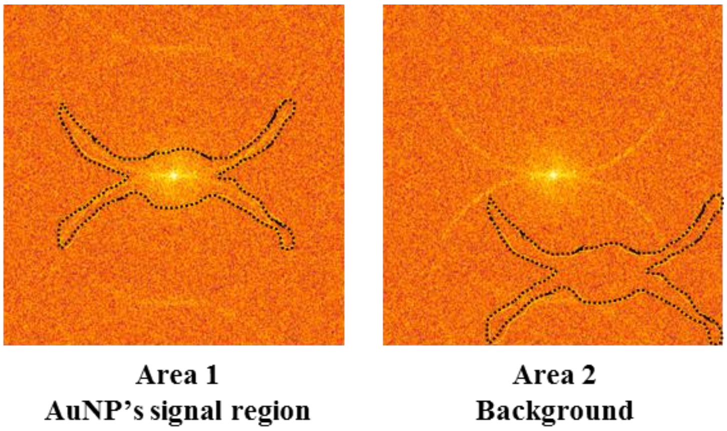


**Figure S4.** The selected region for extracting the signal of AuNP ($I_{NP}$) and background regions ($I_{Background}$) from the spatial FFT of P-EIM images in **Fig. 3*B***. The normalized intensity change is calculated by ($I_{NP}$-$I_{Background}$).

**S5. The P-EIM images of single gold nanoparticle on C8 applied with different amplitudes of potential**

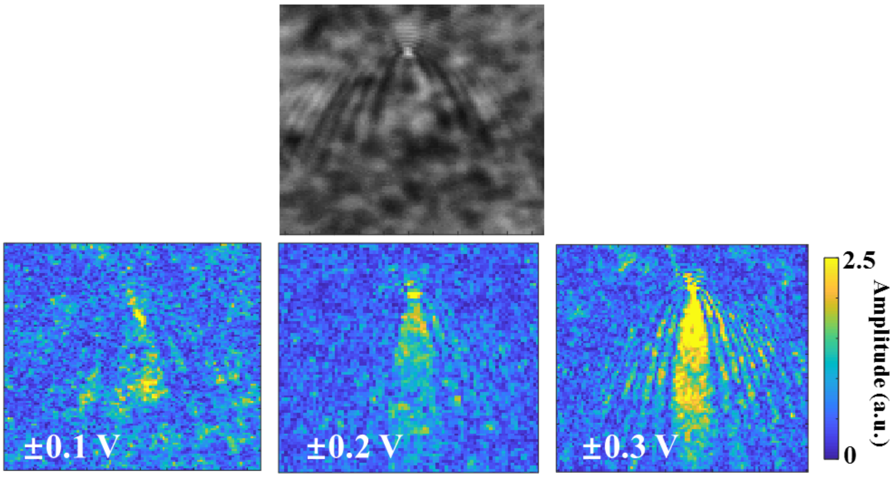


**Figure S5.** The plasmonic image and the corresponding P-EIM images of single gold nanoparticle (Diameter=100 nm) on C8 modified surfaces versus modulation potential with different amplitudes. The frequency of sine-wave potential modulation is 5 Hz. The electrolyte is 0.1 M NaF solution, and the reference electrode is Ag/AgCl.

**S6. The AFM image and surface roughness of n-alkanethiol modified gold surfaces**

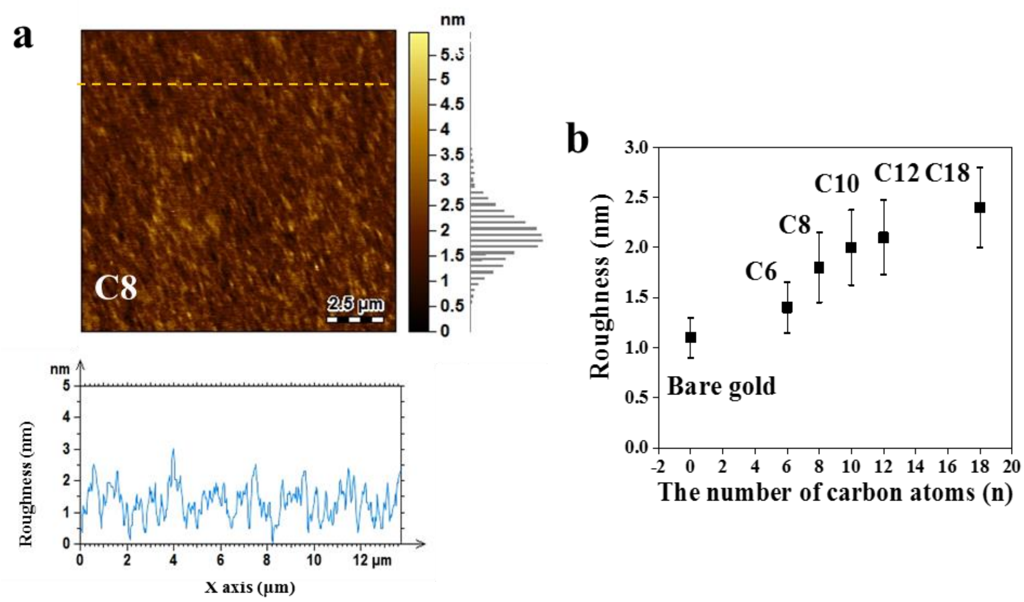


**Figure S6.** (a) The contact-mode AFM image of C8 modified gold electrode surface in deionized water. The scanning rate is 1 line/s. (b) The roughness of thiol modified gold surfaces obtained from AFM images verse the number of carbon atoms of n-alkanethiol.

**S7. The SEM measurement of 100 nm AuNPs**

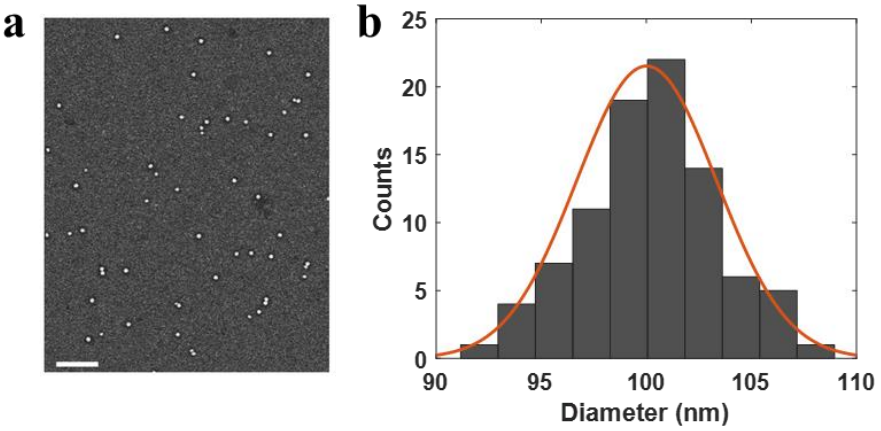


**Figure S7.** (a) SEM image of 100 nm AuNPs on bare gold surface. Scale bare: 1 μm. (b) The diameter distribution and the Gaussian fitting result of the AuNPs from SEM image.

**S8. Statistical analysis of electron neutralization dynamics during individual nanoparticle collision on different surfaces**

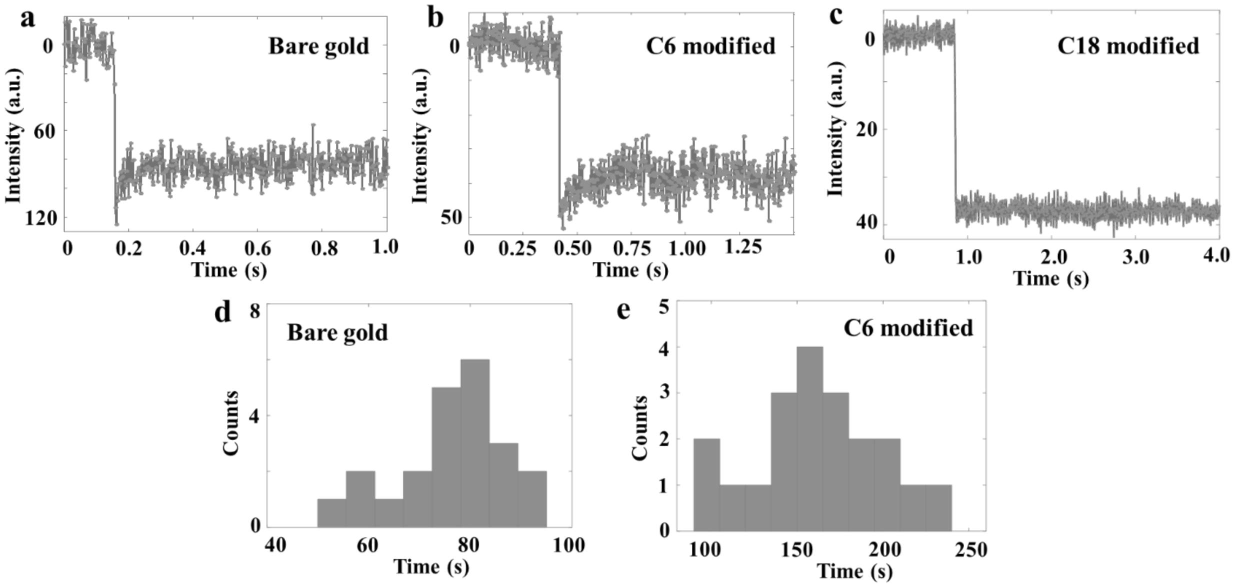


**Figure S8**. (a) Time-lapsed plasmonic intensity curve during single AuNP hitting to the bare gold surface with fast electron neutralization dynamics. (b) Time-lapsed plasmonic intensity curve during single AuNP hitting to the C6 modified surface with slow electron neutralization dynamics. (c) Time-lapsed plasmonic intensity curve during single AuNP hitting to the C18 modified surface without any electron neutralization dynamics. (d) The statistical analysis of time constant for bare gold surface. The average value is 76 ms. (e) The statistical analysis of time constant for C6 modified surface. The average value is 164 ms.

## References

(1) Porter, M. D.; Bright, T. B.; Allara, D. L.; Chidsey, C. E. Spontaneously Organized Molecular Assemblies. *J. Am. Chem. Soc.* **1987**, *109*, 3559-3568.

(2) Rampi, M. A.; Schueller, O. J.; Whitesides, G. M. Alkanethiol Self-Assembled Monolayers as the Dielectric of Capacitors with Nanoscale Thickness. *Appl. Phys. Lett.* **1998**, *72*, 1781-1783.

(3) Liu, R.; Shan, X.; Wang, H.; Tao, N. Plasmonic Measurement of Electron Transfer between a Single Metal Nanoparticle and an Electrode through a Molecular Layer. *J. Am. Chem. Soc.* **2019**, *141*, 11694-11699.
